# Patellar resurfacing in primary total knee arthroplasty is associated with fewer reoperations but comparable clinical and functional outcomes: an umbrella review of meta-analyses

**DOI:** 10.1186/s42836-026-00438-y

**Published:** 2026-10-06

**Authors:** Filippo Migliorini, Nicola Maffulli, Luise Schäfer, Gennaro Pipino, Marcel Betsch, Raju Vaishya

**Affiliations:** 1 Department of Trauma and Reconstructive Surgery, University Hospital of Halle, Martin-Luther University Halle-Wittenberg, 06097 Halle (Saale), Germany; 2 Department of Life Sciences, Health, and Health Professions, Link Campus University, Rome, 00165 Italy; 3 Research Department of Clinical Orthopaedic, Eifelklinik St. Brigida, Kammerbruchstr. 8, Simmerath, 52152 Germany; 4 Department of Trauma and Orthopaedic Surgery, Faculty of Medicine and Psychology, University La Sapienza, 00185 Rome, Italy; 5 School of Pharmacy and Bioengineering, Faculty of Medicine, Keele University, Stoke On Trent, ST4 7QB UK; 6 Centre for Sports and Exercise Medicine, Barts and the London School of Medicine and Dentistry, Mile End Hospital, Queen Mary University of London, London, E1 4DG UK; 7 Department of Orthopaedics, Villa Erbosa Hospital, Milan, 40119 Italy; 8 Department of Orthopaedic, Trauma and Reconstructive Surgery, University Hospital of Erlangen, 91054 Erlangen, Germany; 9 Department of Orthopaedics and Joint Replacement Surgery, Indraprastha Apollo Hospital, Sarita Vihar, New Delhi, 110076 India

**Keywords:** Anterior knee pain, Patellofemoral joint, Revision surgery, Clinical heterogeneity, Evidence synthesis, Implant design, Patient selection

## Abstract

**Background:**

Patellar management in primary total knee arthroplasty (TKA) remains controversial despite numerous clinical trials and meta-analyses. While patellar resurfacing has been advocated to reduce anterior knee pain (AKP) and reoperation rates, reported effects on functional outcomes are inconsistent. This umbrella review synthesised and critically appraised published meta-analyses comparing patellar resurfacing and patellar retention in primary TKA, with a focus on anterior knee pain, reoperation rates, and patient-reported outcome measures (PROMs).

**Methods:**

An umbrella review of meta-analyses was conducted in accordance with 2020 PRISMA guidelines. Meta-analyses comparing patellar resurfacing with patellar retention in primary TKA were eligible. Outcomes of interest were AKP, reoperation rates, knee range of motion, and PROMs. Quantitative synthesis was performed at the meta-analysis level using random-effects models, with heterogeneity assessed using the I^2^ statistic and robustness evaluated through prediction intervals.

**Results:**

Fourteen meta-analyses published between 2011 and 2025 were included. When AKP was analysed using odds ratios (OR), no significant association with patellar resurfacing was observed (OR 1.13, 95% CI 0.71–1.82; I^2^ 88%). In contrast, relative risk (RR)–based analyses demonstrated a significantly reduced risk of AKP with resurfacing (RR 0.75, 95% CI 0.65–0.86; I^2^ 0%). OR–based analyses showed no significant difference (OR 1.44, 95% CI 0.54–3.84; I^2^ 94%), whereas relative risk-based analyses indicated a significantly lower reoperation risk with patellar resurfacing (RR 0.59, 95% CI 0.52–0.67; I^2^ 51%). Functional outcomes showed no clinically relevant differences. Knee Function Score did not differ between groups (MD 0.63, 95% CI −0.42 to 1.68), while Knee Society Score showed a small statistically significant difference (MD 0.92, 95% CI 0.34–1.50) that was not consistently reproducible. No significant differences in postoperative ROM were observed.

**Conclusions:**

Patellar resurfacing in primary TKA reduces the risk of reoperation but does not confer clinically meaningful benefits in PROMs and shows only limited and inconsistent effects on AKP.

**Supplementary Information:**

The online version contains supplementary material available at https://doi.org/10.1186/s42836-026-00438-y.

## Introduction

Total knee arthroplasty (TKA) is an effective treatment for end-stage knee osteoarthritis (OA) [1–3], improving quality of life and enabling most patients to participate in recreational activities [4–7]. Over the past decades, TKA has undergone substantial evolution, with advances in implant design, surgical techniques, and perioperative management contributing to improved clinical outcomes and long-term survivorship [8–11]. However, dissatisfaction persists among 5% to 20% of patients [12–14]. Among the various technical aspects of TKA associated with dissatisfaction, patellar management remains one of the most debated and polarising concerns [15, 16]. This debate is reflected in a survey of 619 surgeons, in which 28% always, 48% selectively, and 24% never resurfaced the patella, with no significant differences according to surgical experience or case volume [17]. Decades after the introduction of modern implant designs, the role of routine patellar resurfacing remains controversial [18–20]. The clinical rationale for patellar resurfacing is intuitive. AKP has long been attributed, at least in part, to patellofemoral biomechanics, cartilage degeneration, and implant-patella interaction [21–24]. Consequently, resurfacing has been advocated as a strategy to reduce postoperative AKP and to minimise the risk of secondary procedures related to patellofemoral symptoms [25, 26]. Conversely, patellar resurfacing is not without drawbacks [25]. Reported complications include patellar fracture, component loosening, extensor mechanism injury, increased surgical time and associated costs [27, 28]. As a result, many surgeons have favoured patellar retention, particularly with improved femoral component design and so-called patellar-friendly trochlear geometries [29, 30]. This divergence in surgical philosophy is evident in both the contemporary literature and national registry data, which demonstrate substantial variability in resurfacing rates across countries and institutions, ranging from 4% in Sweden to 94% in the United States; substantial temporal changes were also observed in the United Kingdom and Australia, with resurfacing rates in Australia exceeding 70% from 2019 onwards [31]. Over the past two decades, an increasing number of comparative clinical studies have investigated outcomes following TKA with and without patellar resurfacing [32–55]. These primary studies, however, have yielded heterogeneous and sometimes conflicting results, particularly regarding AKP and patient-reported outcome measures (PROMs). To address this inconsistency, multiple systematic reviews and meta-analyses have been published [19, 25, 56–67]. While many of these meta-analyses suggest a reduced risk of reoperation associated with patellar resurfacing, their conclusions regarding PROMs, pain relief, and functional outcomes are less consistent. More recent analyses have introduced additional complexity, including implant design, follow-up duration, selective resurfacing strategies, and economic considerations [67–69]. Paradoxically, the increasing number of meta-analyses has perpetuated uncertainty. Substantial heterogeneity in inclusion criteria, methodological rigour, statistical methods, and outcome definitions complicates the interpretation of the evidence and precludes clear overarching conclusions. Despite high-level evidence, no previous umbrella review that systematically synthesises and critically appraises the available meta-analyses has been published. Therefore, an umbrella review was conducted to analyse published meta-analyses comparing patellar resurfacing versus patellar retention in primary TKA. The outcomes of interest were AKP, reoperation rates, range of motion (ROM), and PROMs.

## Methods

### Eligibility criteria

All meta-analyses comparing patellar resurfacing with patellar retention in primary TKA were considered. Only studies published in peer-reviewed journals were considered. According to the authors’ language capabilities, articles in English, German, Italian, French, and Spanish were eligible. Only studies with Level I–III evidence, according to the Oxford Centre of Evidence-Based Medicine [5], were included. Clinical studies, systematic reviews without meta-analysis, narrative reviews, opinions, letters, and editorials were not considered, nor were animal, in vitro, biomechanical, computational, or cadaveric studies. Missing quantitative data for outcomes of interest warranted exclusion of the study.

### Search strategy

This study was conducted according to the 2020 Preferred Reporting Items for Systematic Reviews and Meta-Analyses (PRISMA) guidelines [6]. The following PICOD framework was used:

- P (problem): resurfacing the patella in primary TKA;
- I (intervention): patella resurfacing;
- C (comparator): patella retention;
- O (outcomes): PROMs, anterior knee pain, reoperations;
- D (design): meta-analysis.

In January 2026, the following databases were accessed: PubMed, Scopus, and Web of Science. No time constraint was set for the search. The Medical Subject Headings (MeSH) used for the database search are reported in Table 1.

**Table 1:** Strings used for the search in each database

Database	Search String
PubMed	((“Arthroplasty, Replacement, Knee”[Mesh] OR “knee arthroplasty”[tiab] OR “knee replacement”[tiab] OR TKA[tiab] OR TKR[tiab]) AND (patella*[tiab] OR “Patella”[Mesh] OR patellofemoral[tiab]) AND (resurfac*[tiab] OR “patellar resurfacing”[tiab] OR “patella resurfacing”[tiab] OR “patellar replacement”[tiab] OR retain*[tiab] OR “patellar retention”[tiab] OR “patella retention”[tiab] OR nonresurfac*[tiab] OR “non resurfacing”[tiab] OR “not resurfaced”[tiab]) AND (“Meta-Analysis”[Publication Type] OR “Systematic Review”[Publication Type] OR meta-analysis[tiab] OR systematic review[tiab] OR pooled[tiab])) NOT (“Arthroplasty, Replacement, Knee, Revision”[Mesh] OR revision[tiab] OR re-revision[tiab])
Scopus	TITLE-ABS-KEY((“total knee arthroplasty” OR “total knee replacement” OR TKA OR TKR OR “knee arthroplasty” OR “knee replacement”) AND (patella* OR patellofemoral) AND (resurfac* OR “patellar resurfacing” OR “patella resurfacing” OR “patellar replacement” OR retain* OR “patellar retention” OR “patella retention” OR nonresurfac* OR “non resurfacing” OR “not resurfaced”) AND (“systematic review” OR “meta-analysis” OR metaanalysis)) AND (LIMIT-TO(DOCTYPE, “re”))
WoS	TS = ((“total knee arthroplasty” OR “total knee replacement” OR TKA OR TKR OR “knee arthroplasty” OR “knee replacement”) AND (patella* OR patellofemoral) AND (resurfac* OR “patellar resurfacing” OR “patella resurfacing” OR “patellar replacement” OR retain* OR “patellar retention” OR “patella retention” OR nonresurfac* OR “non resurfacing” OR “not resurfaced”) AND (“systematic review” OR “meta-analysis” OR metaanalysis)) NOT TS = (revision OR re-revision OR “revision arthroplasty”)

WoS: Web of Science

### Selection and data collection

Two authors (G.P. & M.B.) independently conducted the database search. All retrieved records were initially assessed at the title level, and abstracts were subsequently reviewed when deemed potentially relevant. Full-text articles were then obtained for studies addressing the topic of interest. Publications for which the full text could not be retrieved were excluded from further evaluation. In addition, the reference lists of all eligible full-text articles were manually screened to identify further pertinent studies. Any discrepancies between the two reviewers were discussed until consensus was reached; unresolved disagreements were adjudicated by a third author (N.M.).

### Data items

Two authors (G.P. & M.B.) independently performed data extraction. Data collected comprised bibliographic details, study characteristics, sample size, intervention and comparator information comparing patellar resurfacing with patellar retention, and all reported clinical outcomes, including AKP, functional scores, reoperation rates, complications, and ROM. Data were extracted in Microsoft Office Excel version 16.72 (Microsoft Corporation, Redmond, USA). When outcome definitions differed across the included meta-analyses, data were extracted according to the definitions provided by the original authors. No post hoc harmonisation or reclassification of outcomes was undertaken, and these differences were considered a potential source of clinical heterogeneity during interpretation of the findings.

### Methodological quality assessment

Two authors (L.S. & G.P.) performed the methodological quality assessment using the critical appraisal tool for systematic reviews that includes randomised or non-randomised studies of healthcare interventions (AMSTAR-2) [7]. The assessment was conducted independently by both reviewers, and any discrepancies were discussed until agreement was reached. If consensus could not be achieved, a third senior author (N.M.) was consulted for final adjudication. AMSTAR-2 consists of 16 item-level questions that address key aspects of conducting systematic reviews. Each item was assessed according to predefined criteria, indicating whether the methodological standard was fully met (“yes”), partially met (“partial yes”), or not met. The appraisal focused on core methodological domains, including the adequacy of the search strategy, the assessment of risk of bias in the included studies, the appropriateness of the statistical methods, and the consideration of potential sources of bias when interpreting the results. In accordance with AMSTAR-2 recommendations, no overall numerical score was calculated. Instead, overall confidence in each meta-analysis’s findings was assessed based on the presence of critical and non-critical methodological limitations. Meta-analyses without critical weaknesses were considered to provide moderate to high confidence, whereas the presence of one or more critical weaknesses resulted in low or critically low confidence.

### Statistical analyses

The statistical analyses were conducted by the first author (F.M.) using R (R Foundation for Statistical Computing, Vienna, Austria), primarily employing the meta package. All quantitative analyses were performed at the meta-analysis level, in accordance with established methodological recommendations for umbrella reviews. For each outcome of interest, pooled effect estimates and corresponding 95% confidence intervals (CIs) reported by the individual meta-analyses were extracted and re-analysed. Binary outcomes, including anterior knee pain and reoperation, were synthesised using relative risks or odds ratios as reported in the original meta-analyses. In contrast, continuous outcomes were analysed using mean differences. Effect estimates for ratio measures were analysed on the logarithmic scale and subsequently back-transformed for presentation, while continuous outcomes were analysed on their original scale. For each outcome, a random-effects model was applied, regardless of the statistical approach used in the original meta-analyses. Given the expected clinical and methodological heterogeneity across reviews, including differences in study selection criteria, patient populations, follow-up duration, outcome definitions, and analytical methods, a random-effects approach was considered the most appropriate framework for umbrella-level synthesis. This strategy also ensured methodological consistency across all analyses and accounted for variability arising both within and between meta-analyses. The restricted maximum-likelihood method was used to estimate the between–meta-analysis variance (τ^2^). Statistical heterogeneity was quantified using the I^2^ statistic, with higher values indicating increasing inconsistency across meta-analyses. In addition to conventional pooled estimates and confidence intervals, prediction intervals were calculated for all outcomes with sufficient data, providing an estimate of the range within which the true effect of a future meta-analysis is expected to lie. Prediction intervals were considered a key component of the robustness assessment, as they incorporate both within- and between–meta-analysis uncertainty and offer complementary information to the 95% CI. Sensitivity analyses were conducted by stratifying binary outcomes by the effect measure used, comparing results from relative risk (RR) and odds ratio (OR) analyses. Differences in pooled estimates, heterogeneity, and prediction intervals across these analyses were examined to assess the stability and reproducibility of the findings. A two-sided significance level of 0.05 was adopted throughout. To quantify the degree of overlap among primary studies shared across the included meta-analyses, the corrected covered area (CCA) was calculated as described by Pieper et al. [8]. A citation matrix was constructed by cross-tabulating every primary study included across the 14 meta-analyses. Multiple reports representing the same underlying patient cohort, including sequential follow-up publications and duplicate/e-publication reports, were collapsed into a single cohort. The CCA was calculated as (N − r)/[(r × c) − r], where N is the total number of primary study occurrences across all meta-analyses after cohort-level deduplication, r is the number of unique primary cohorts, and c is the number of included meta-analyses.

## Results

### Search results

**Fig. 1:**
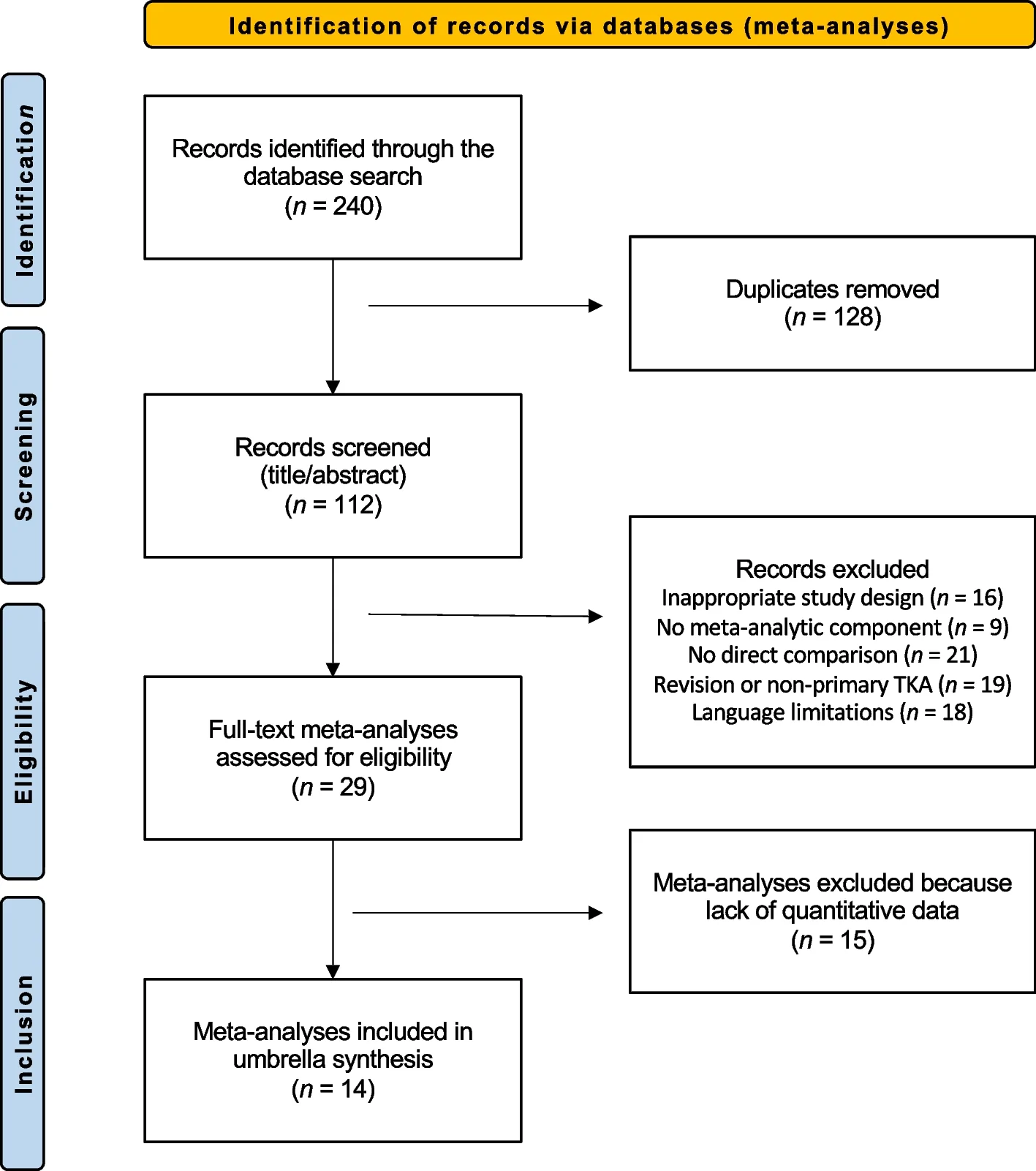
PRISMA flowchart of the search results

The literature search identified 240 records across the selected databases. After 128 duplicates were removed, 112 records underwent title and abstract screening. Of these, 83 articles were excluded because they did not meet the predefined inclusion criteria, primarily because of inappropriate study design (*N* = 16), absence of a meta-analytic component (*N* = 9), no direct comparison between patellar resurfacing and patellar retention in primary TKA (*N* = 21), focus on revision TKA or non-primary procedures (*N* = 19), and language limitations (*N* = 18). Twenty-nine full-text articles were assessed for eligibility. Fifteen reviews were subsequently excluded because they did not report quantitative outcome data suitable for umbrella-level synthesis. Ultimately, 14 meta-analyses fulfilled all eligibility criteria and were included in the final quantitative umbrella synthesis. The study selection process is illustrated in the PRISMA flow diagram (Fig. 1).

### Methodological quality assessment

**Fig. 2:**
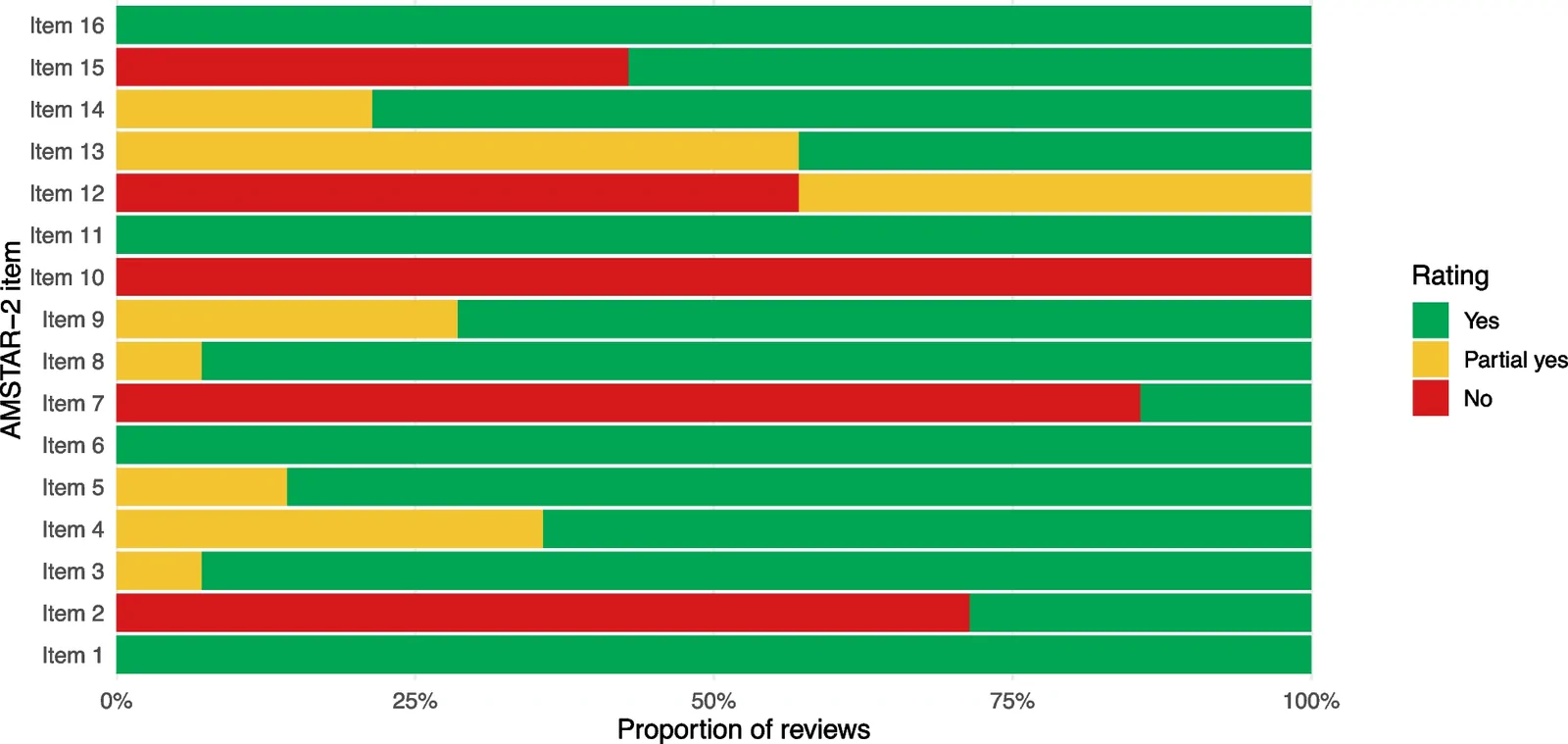
The AMSTAR-2 graph. The distribution of “Yes”, “Partial yes”, and “No” ratings is shown for each of the 16 AMSTAR-2 items. Items address key methodological aspects including PICO formulation and protocol registration (Items 1–2), literature search and study selection (Items 3–5), data extraction and study description (Items 6–8), risk of bias assessment and synthesis methods (Items 9–13), and the assessment of heterogeneity, publication bias, and conflicts of interest (Items 14–16). A detailed description of the AMSTAR-2 items is provided in Supplementary Table S1

The methodological quality of the included systematic reviews was assessed using the AMSTAR-2 tool [70]. Across the majority of included reviews, high adherence to fundamental methodological items was observed, including a PICO-based formulation of the research question, comprehensive literature search strategies, duplicate study selection and data extraction, as well as adequate reporting of study characteristics. These items were predominantly rated as “Yes”, indicating a generally robust methodological framework among most included reviews. Notably, Grela et al. [56] consistently fulfilled nearly all AMSTAR-2 items, including the predefined critical domains, resulting in a high-confidence methodological profile across the full item set. In contrast, Tang et al. [57] demonstrated multiple deficiencies across several key items, particularly those related to protocol registration, transparency of excluded studies, and the assessment and consideration of risk of bias and publication bias, leading to a critically low-confidence profile at the item level (Supplementary Fig. S1). While most reviews showed satisfactory performance in core methodological domains, variability was observed in items addressing protocol registration, the justification of excluded studies, and the investigation of publication bias, which accounted for the majority of “Partial yes” and “No” ratings across the dataset. The item-level distribution of ratings across the 16 AMSTAR-2 items served as the basis for deriving overall confidence ratings for the included meta-analyses. 7.1% (1 of 14) of the included meta-analyses were judged to provide high confidence in their findings, whereas 85.7% (12 of 14) were rated as low confidence and 7.1% (1 of 14) as critically low confidence. The low confidence ratings were mainly attributable to methodological shortcomings, including the absence of protocol registration, lack of transparent reporting of excluded studies, and insufficient integration of risk-of-bias assessments into the quantitative synthesis. Overall, adherence to individual AMSTAR-2 items was generally high across the included reviews. Nevertheless, weaknesses in specific critical domains, particularly protocol registration, resulted in predominantly low overall confidence ratings and warrant caution when interpreting the reviews’ conclusions. The item-level distribution of ratings across the 16 AMSTAR-2 items is illustrated in Fig. 2.

### Generalities of included studies

Fourteen meta-analyses published between 2011 and 2025 were included in this umbrella review. Most were classified as level I evidence and were published in leading orthopaedic journals. The number of primary studies per meta-analysis ranged from 10 to 50, encompassing patient populations from approximately 1,000 to more than 500,000 individuals (Table 2). AKP was assessed in all included meta-analyses, while functional outcomes were most commonly evaluated using the Knee Society Score (KSS) and Knee Function Score (KFS). Additional PROMs and secondary endpoints, such as reoperation, complications, infections, and ROM, were reported less consistently across studies, reflecting variability in outcome selection and methodological focus.

**Table 2:** General characteristics of the included meta-analyses

Author and year	Journal	LoE	Studies (*n*)	Patients (*n*)	Outcome of interest
Arirachakaran et al., 2015 [58]	*Knee Surg Sports Traumatol Arthrosc*	I	18	3,449	AKP, KSS, KFS, VAS, Infections, Complications, Reoperations
Chen et al., 2013 [59]	*Int Orthop*	I	14	1,725	AKP, KSS, KFS, KPS, reoperation
Chen et al., 2021 [19]	*J Arthroplasty*	I	32	6,887	AKP, KSS, KFS, Reoperation, ROM, OKS, KOOS
Fu et al., 2011 [60]	*Knee Surg Sports Traumatol Arthrosc*	II	10	1,003	Reoperation, complications, AKP, KFF, KSS
Grela et al., 2022 [56]	*BMC Musculoskelet Disord*	I	32	5,499	AKP, KSS, KFS, Reoperation, ROM, OKS, KOOS, Complications, VAS
He et al., 2011 [61]	*Knee*	I	16	3,034	AKP, KSS, KFS, KPS, reoperation
Migliorini et al., 2019 [25]	*Arch Orthop Trauma Surg*	II	31	4,132	AKP, KSS, KFS, KPS, ROM reoperation
Pavlou et al., 2011 [62]	*J Bone Joint Surg Am*	I	18	7,075	AKP, KFS, reoperation
Pilling et al., 2012 [63]	*J Bone Joint Surg Am*	I	16	3,465	KSS, AKP, Reoperation, Complications, Surgical time, Infections
Simpson et al., 2023 [64]	*Knee*	I	32	4,085	AKP, KSS, KFS, Reoperation, OKS, Complications
Tang et al., 2018 [57]	*J Arthroplasty*	I	20	2,573	AKP, KSS, KFS, Infections, Reoperations
Tang et al., 2023 [65]	*Orthop Surg*	I	50	5,586	AKP, KSS, KFS, Reoperation, ROM, OKS, KOOS, WOMAC, Surgical Time, Complications
Teel et al., 2019 [66]	*J Arthroplasty*	I	20	3,162	AKP, KSS, KFS, Infections, Reoperations, Complications
Usman et al., 2025 [67]	*J Orthop Surg Res*	III	49	54,6566	AKP, KSS, OKS, VAS, WOMAC, ROM

AKP: anterior knee pain; KSS: Knee Society Score; KFS: Knee Function Score; KPS: Knee Pain Score; KFF: Knee Flexion Function; VAS: visual analogue scale; ROM: range of motion; OKS: Oxford Knee Score; KOOS: Knee Injury and Osteoarthritis Outcome Score; WOMAC: Western Ontario and McMaster Universities Osteoarthritis Index; LoE: level of evidence

### Anterior knee pain

**Fig. 3:**
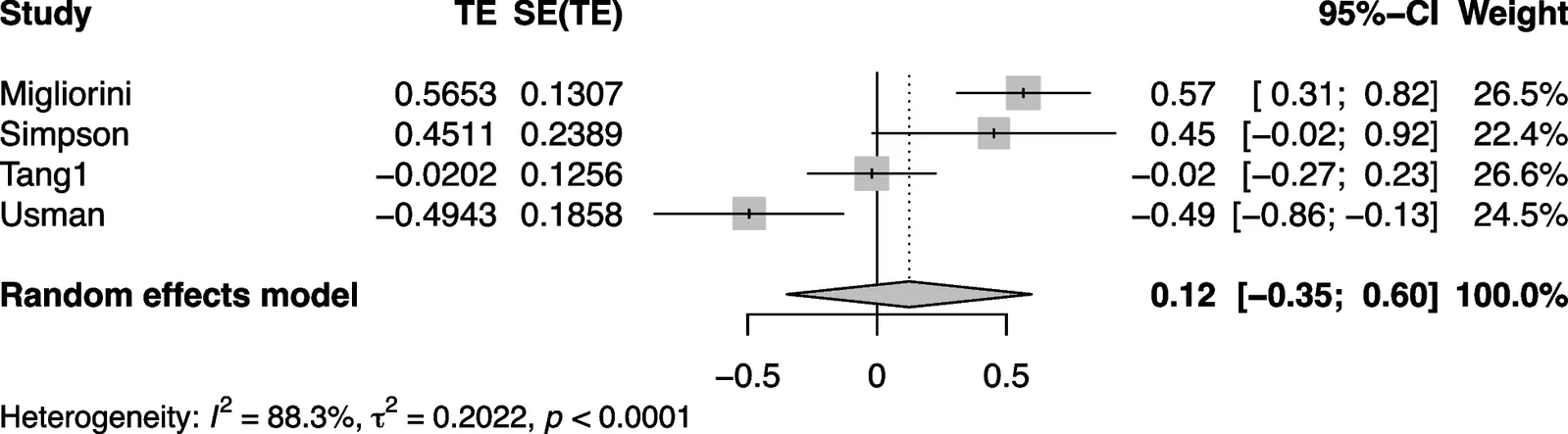
Umbrella results of meta-analyses investigating AKP using the OR effect measure. Individual effect estimates, 95% confidence intervals (CIs), and study weights are displayed within the forest plot

Across umbrella analyses, discrepant results emerged according to the effect measure used. When pooled as OR, no statistically significant association was observed between patellar resurfacing and the risk of AKP (OR = 1.13, 95% CI = 0.71–1.82; k = 4; Fig. 3). Substantial heterogeneity was detected (I^2^ = 88%), and the prediction interval was extremely wide (0.22–5.74), indicating marked uncertainty and limited reproducibility of the pooled estimate.

**Fig. 4:**
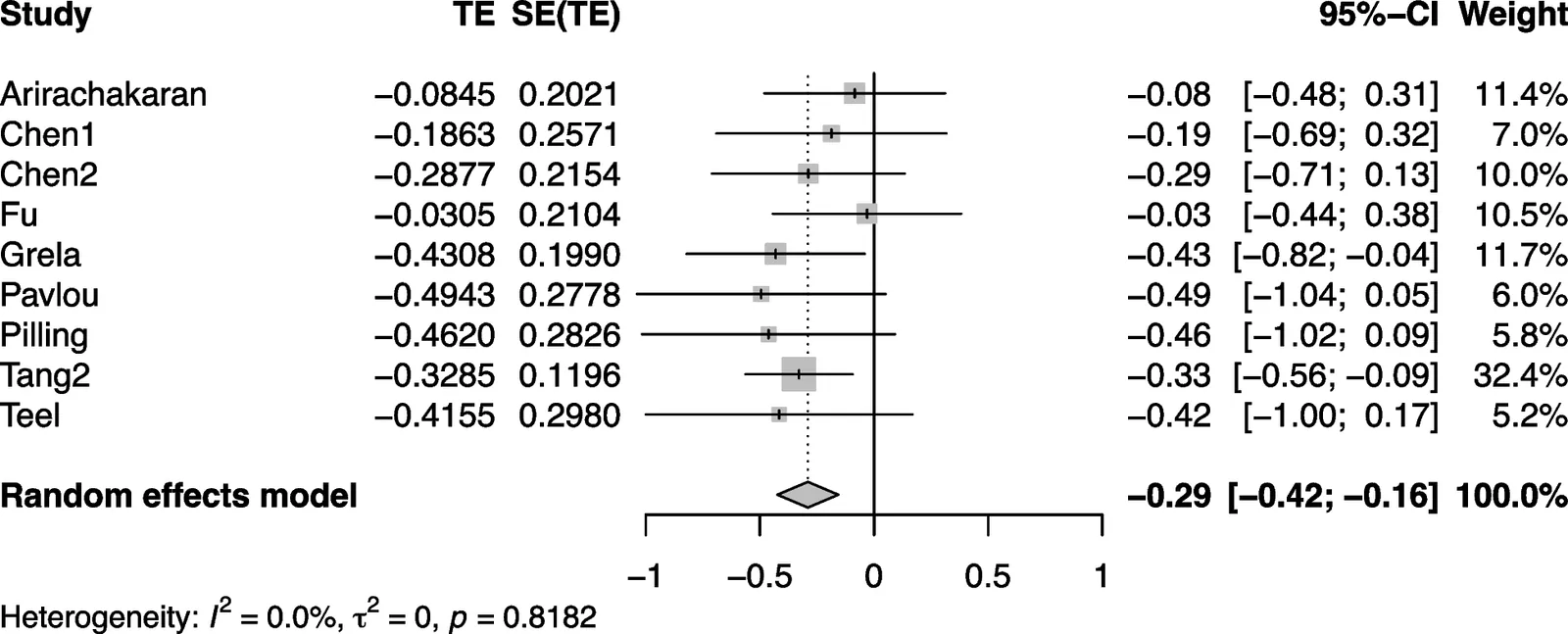
Umbrella results of meta-analyses investigating AKP using the RR. Individual effect estimates, 95% confidence intervals (CIs), and study weights are displayed within the forest plot

Conversely, when the same outcome was synthesised using RR, patellar resurfacing was associated with a significantly lower risk of AKP (RR 0.75, 95% CI 0.65–0.86; k = 9; Fig. 4). No heterogeneity was observed (I^2^ = 0%), and the prediction interval remained narrow and entirely below unity (0.64–0.88), supporting the robustness and consistency of this finding.

### Reoperation rates

**Fig. 5:**
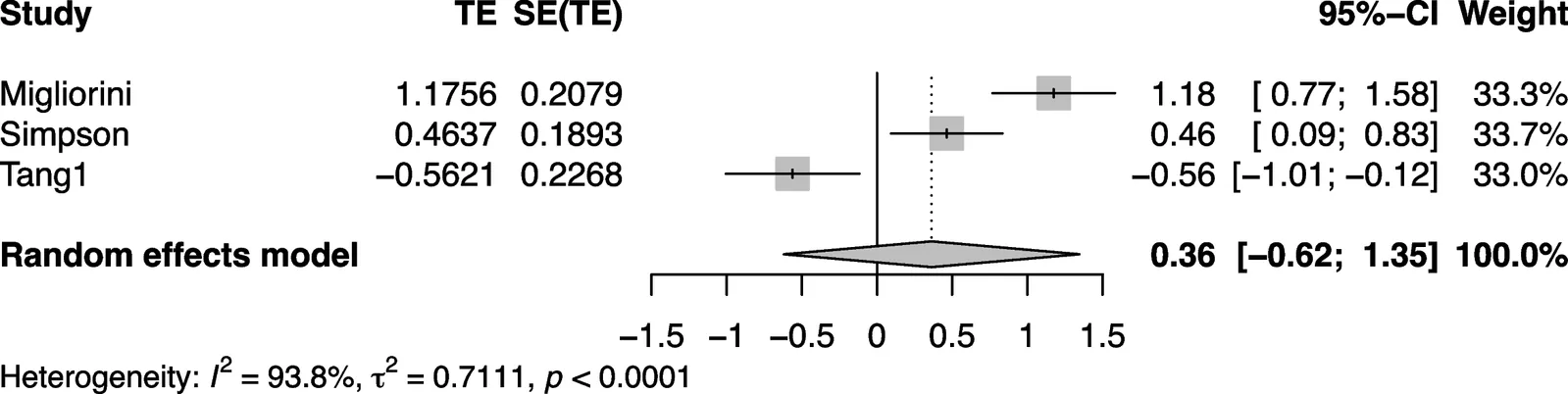
Umbrella results of meta-analyses investigating the reoperation rate using the OR. Individual effect estimates, 95% confidence intervals (CIs), and study weights are displayed within the forest plot

Umbrella analyses of reoperation rates yielded analogous discrepancies. When expressed as OR, no significant association was observed (OR 1.44, 95% CI 0.54–3.84; k = 3; Fig. 5), with substantial heterogeneity (I^2^ = 94%) and an excessively wide prediction interval (0.02–97.9), precluding meaningful clinical interpretation.

**Fig. 6:**
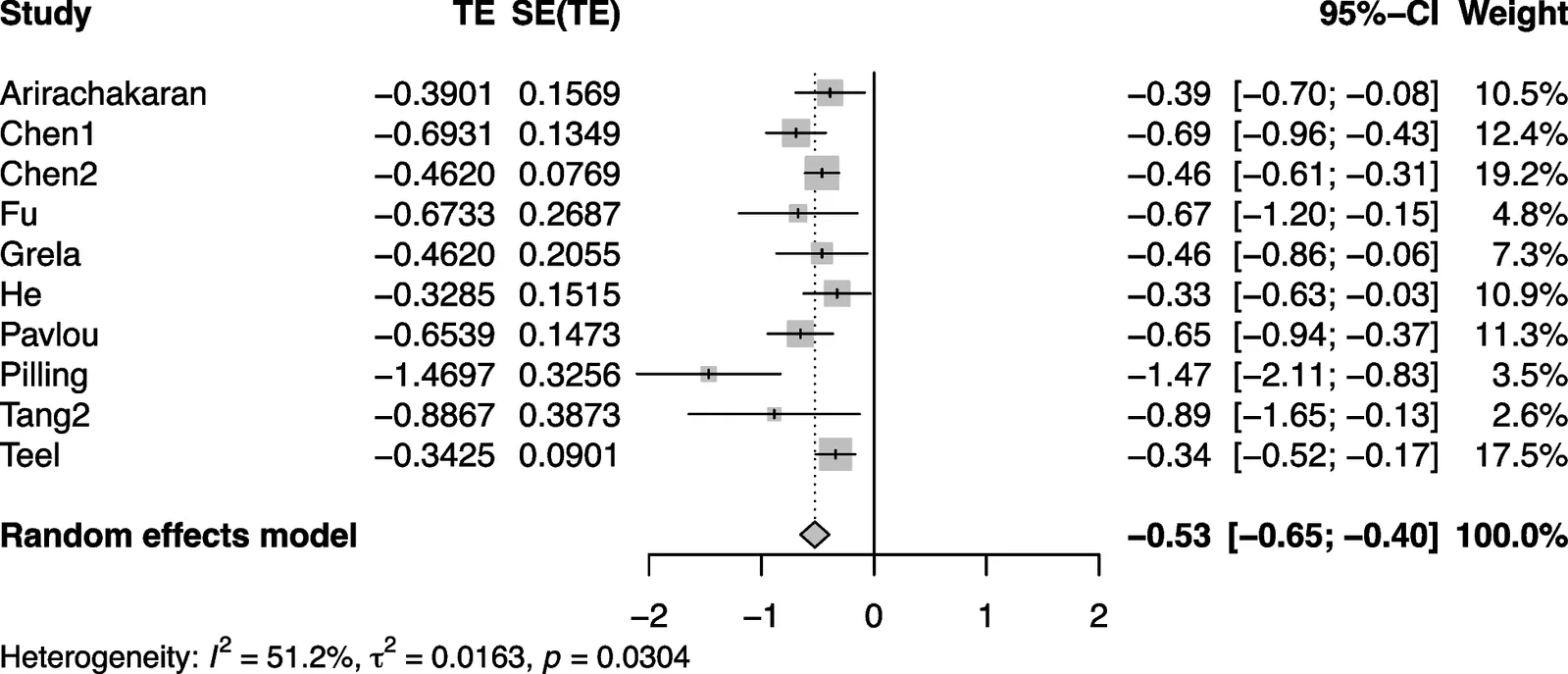
Umbrella results of meta-analyses investigating the reoperation rate using the RR. Individual effect estimates, 95% confidence intervals (CIs), and study weights are displayed within the forest plot

In contrast, analyses based on RR demonstrated a significantly reduced risk of reoperation associated with patellar resurfacing (RR 0.59, 95% CI 0.52–0.67; k = 10; Fig. 6). Heterogeneity was moderate (I^2^ = 51%), and the prediction interval (0.43–0.82) remained fully below unity, indicating a clinically relevant and reproducible effect.

### Functional outcomes and range of motion

**Fig. 7:**
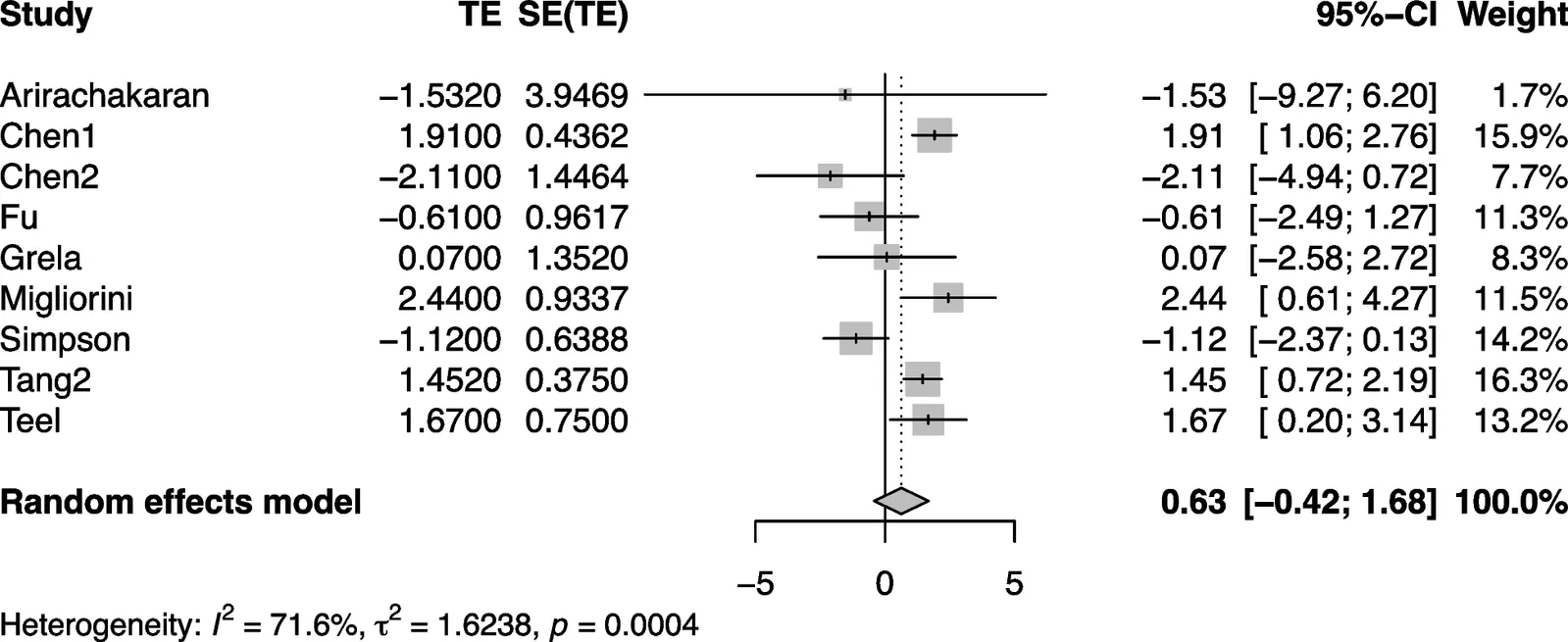
Umbrella results of meta-analyses investigating the KFS. Individual effect estimates, 95% confidence intervals (CIs), and study weights are displayed within the forest plot

For knee function, the pooled mean difference in the KFS did not reach statistical significance (MD 0.63, 95% CI − 0.42 to 1.68; k = 9; Fig. 7), with substantial heterogeneity (I^2^ = 72%) and a wide prediction interval (− 2.56 to 3.82).

**Fig. 8:**
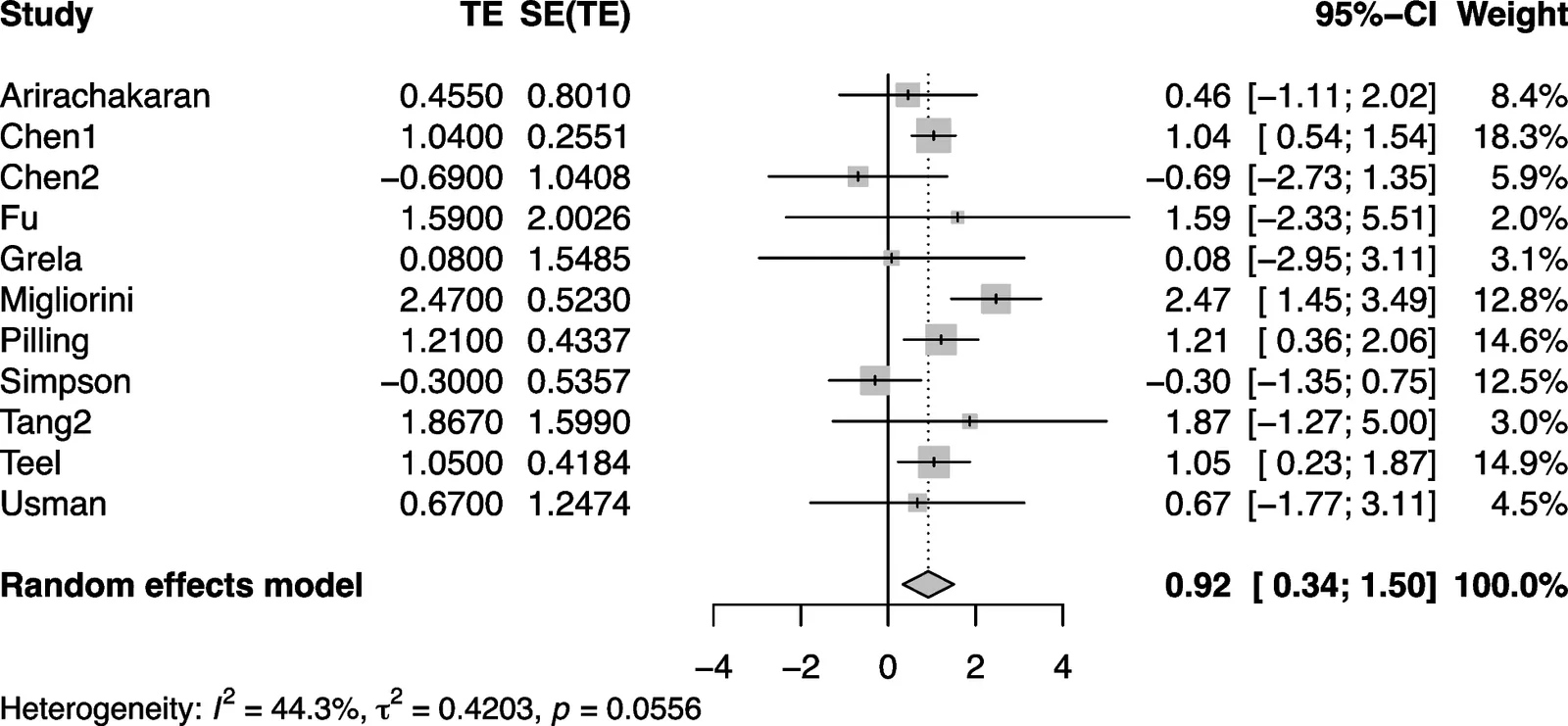
Umbrella results of meta-analyses investigating the KSS. Individual effect estimates, 95% confidence intervals (CIs), and study weights are displayed within the forest plot

The KSS showed a small but statistically significant pooled effect (MD 0.92, 95% CI 0.34–1.50; k = 11; Fig. 8), accompanied by moderate heterogeneity (I^2^ = 44%). However, the prediction interval crossed the null effect range (− 0.67 to 2.51), suggesting that this benefit may not be consistently replicated in future settings.

**Fig. 9:**
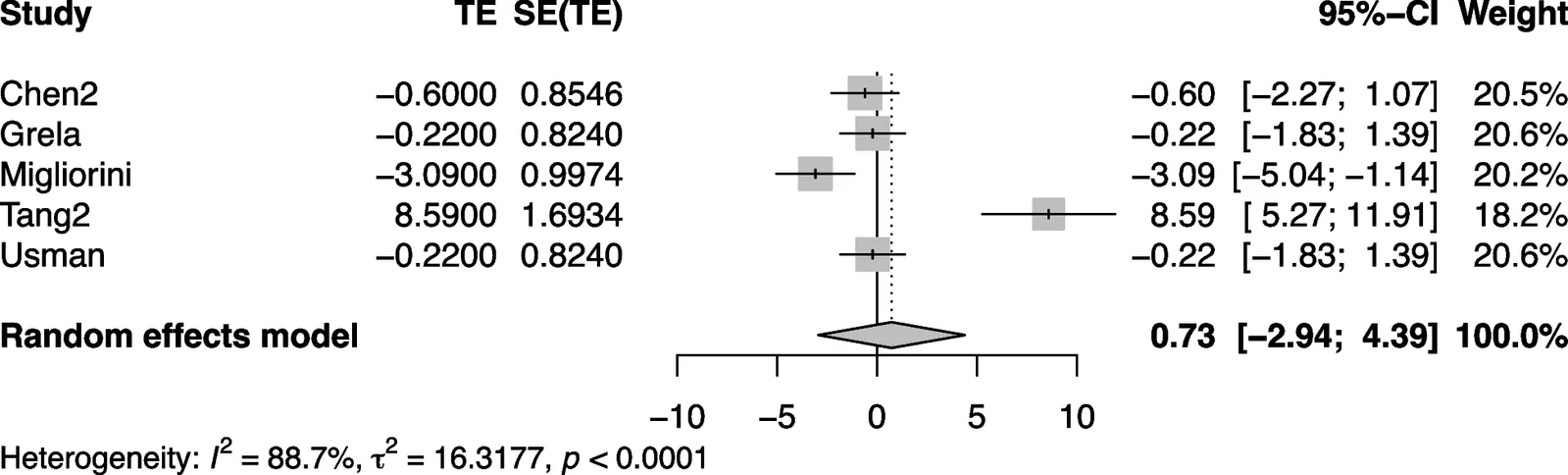
Umbrella results of meta-analyses investigating the ROM. Individual effect estimates, 95% confidence intervals (CIs), and study weights are displayed within the forest plot

No significant difference in postoperative knee ROM was observed (MD 0.73, 95% CI − 2.94 to 4.39; k = 5; Fig. 9). Heterogeneity was high (I^2^ = 89%), and the prediction interval was extremely broad (− 11.6 to 13.1), indicating substantial variability and lack of reliable evidence for a meaningful effect.

### Sensitivity analysis

Sensitivity analyses stratified by effect measures revealed substantial differences in both magnitude and robustness of the pooled estimates. For AKP, odds ratio-based umbrella analyses showed no significant association (OR 1.13, 95% CI 0.71–1.82; k = 4), with very high heterogeneity (I^2^ = 88%) and a wide prediction interval (0.22–5.74). In contrast, relative risk-based analyses demonstrated a significantly reduced risk (RR 0.75, 95% CI 0.65–0.86; k = 9), with no heterogeneity (I^2^ = 0%) and a narrow prediction interval fully below unity (0.64–0.88). A similar pattern was observed for reoperation rates. Odds ratio-based analyses were characterised by non-significant results (OR 1.44, 95% CI 0.54–3.84; k = 3), extreme heterogeneity (I^2^ = 94%), and an excessively wide prediction interval (0.02–97.9). Conversely, relative risk-based analyses indicated a significantly lower risk of reoperation (RR 0.59, 95% CI 0.52–0.67; k = 10), with moderate heterogeneity (I^2^ = 51%) and a prediction interval remaining below unity (0.43–0.82).

### Citation overlap across included meta-analyses

**Fig. 10:**
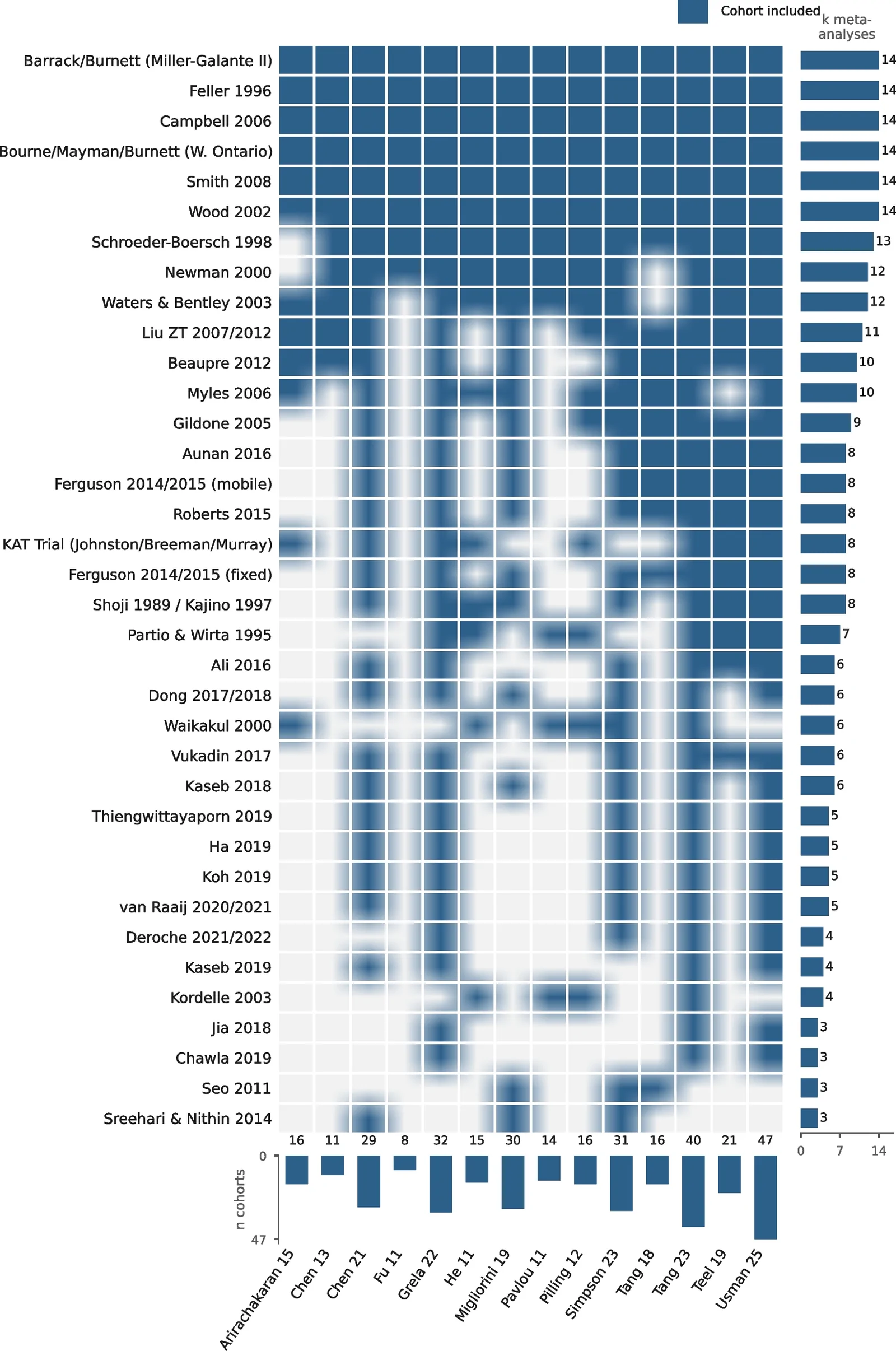
Citation overlap across included meta-analyses

Across the 14 included meta-analyses, 367 raw primary-study citations were identified. After collapsing multiple reports representing the same underlying patient cohort, 326 study–meta-analysis occurrences corresponding to 71 unique primary cohorts remained in the citation matrix. The corrected covered area was 27.6%, indicating a very high degree of overlap among the primary studies informing the included meta-analyses (Fig. 10).

## Discussion

The findings of this umbrella review suggest that the primary benefit of patellar resurfacing in primary TKA lies not in measurable improvements in function or PROMs, but in reduced reoperation risk. The consistent association between resurfacing and lower reoperation rates across multiple meta-analyses suggests this effect is unlikely to be incidental or driven by isolated study designs. Instead, it points to a structural difference in how patellofemoral problems evolve when the native patella is retained. From a clinical standpoint, these findings suggest that patellar resurfacing may function as a preventive strategy against late patellofemoral failure, rather than enhancing early postoperative recovery or perceived knee function. The absence of a uniform, clinically meaningful effect on AKP rate warrants careful interpretation. AKP after TKA is multifactorial and influenced by extensor mechanism biomechanics, quadriceps function, component positioning and rotation, patellar height, soft-tissue balance, and central pain modulation, as well as by local patellofemoral cartilage pathology [21, 71, 72]. Within this context, the proportion of patients in whom AKP is predominantly driven by isolated patellar cartilage degeneration or articular incongruity is likely limited [73]. Patellar resurfacing primarily addresses cartilage-related nociceptive sources by modifying contact mechanics and eliminating degenerated articular surfaces, thereby reducing focal stress and stimulation of the subchondral bone [67, 74]. However, when AKP originates from maltracking secondary to rotational errors, altered extensor mechanism kinematics, or non-mechanical pain pathways, resurfacing is mechanistically unlikely to provide substantial benefit [56, 75]. This explains why the analgesic effect of resurfacing is modest and inconsistently observed across meta-analyses. The small signal in favour of resurfacing reported in some studies likely reflects a restricted subgroup of patients in whom patellofemoral cartilage pathology represents the dominant pain generator [19, 56, 58, 60]. When pooled across heterogeneous populations and non-stratified indications, this effect becomes diluted, contributing to inter-study variability and limited overall effect sizes. This observation underscores the importance of appropriate patient selection, as the benefit of resurfacing appears indication-dependent rather than universal. Although contemporary patellar-friendly designs have been proposed as a potential explanation for the reduced incidence of patellofemoral complications in recent series, the included meta-analyses did not consistently stratify outcomes according to implant design. Therefore, the influence of prosthesis generation on the observed results remains speculative and warrants dedicated investigation [76, 77]. While these advances likely attenuate the incremental benefit of resurfacing at the population level, they do not eliminate patellofemoral pathology, which may account for the persistent, albeit small, advantage observed in selected outcomes [78]. The discrepancy between the RR- and OR-based analyses for AKP likely reflects methodological rather than clinical differences. The OR-based synthesis included fewer meta-analyses and showed substantial heterogeneity, whereas the RR-based analysis incorporated a larger body of evidence with no detectable heterogeneity. Therefore, the divergent findings are more likely attributable to differences in statistical properties and between-review variability than to genuinely conflicting clinical effects. However, the low heterogeneity and narrow prediction intervals observed in the RR-based analyses should be interpreted in the context of the substantial overlap between meta-analyses, as their apparent consistency partly reflects the repeated inclusion of the same historical trials rather than fully independent replication across distinct evidence bases.

Selective patellar resurfacing is conceptually appealing, as it aims to align surgical decision-making with patellofemoral biomechanics and cartilage pathology [79, 80]. However, current evidence indicates that this strategy has not been adequately evaluated within RCTs [61, 81]. Most RCTs compared routine resurfacing with systematic non-resurfacing without stratifying patients according to patellar cartilage status, morphology, or extensor mechanism characteristics, thereby limiting the ability to detect indication-specific benefits [58, 60, 82]. As a consequence, any potential advantage of resurfacing in appropriately selected patients is likely diluted when analysed across heterogeneous, non-stratified cohorts. Moreover, selective resurfacing is inherently surgeon-dependent and lacks standardised criteria, making it difficult to test within rigid trial designs and even more challenging to synthesise in meta-analyses. The absence of a strong signal in favour of selective resurfacing should therefore be interpreted as a methodological limitation rather than evidence of biomechanical equivalence between strategies.

Several limitations must be acknowledged when interpreting the findings of this umbrella review, largely from substantial heterogeneity across the included meta-analyses and, indirectly, across the primary studies on which they are based. A major source of variability relates to differences in study eligibility and level of evidence. Some meta-analyses restricted inclusion to RCTs, whereas others incorporated observational studies and registry data. Sample size and study overlap represent an additional source of heterogeneity. Earlier meta-analyses were based on relatively small numbers of trials and limited aggregated cohorts, making their results more sensitive to the influence of individual studies. More recent analyses included more knees but often relied on substantially overlapping trial populations, formally confirmed by a corrected covered area of 29.9% across the 14 included meta-analyses, a level classified as high overlap according to Pieper et al. [83]. A small set of historical RCTs from the 1990 s and early 2000 s, several reporting fewer than 100 knees, were repeatedly re-pooled across nearly every meta-analysis identified, meaning that the apparent precision gained from accumulating successive meta-analyses over the past three decades is, to a substantial extent, illusory: much of the “new” evidence consists of the same primary cohorts re-analysed under different statistical models and outcome groupings rather than genuinely independent confirmation. This repeated pooling of the same primary studies, combined with differences in data extraction, outcome definitions, and statistical modelling, contributes to variability across meta-analyses and should temper confidence in the apparent consistency of pooled effect estimates, particularly for reoperation, where the same handful of trials dominate the evidence base across all included reviews. Follow-up duration also differed markedly between meta-analyses and has important implications for interpretation. Reoperation is a time-dependent outcome, and short-term follow-up predominantly captures early patellofemoral symptoms and secondary resurfacing. In contrast, longer follow-up incorporates additional failure mechanisms that may be unrelated to patellar management. Some meta-analyses attempted to stratify results by follow-up length, while others pooled disparate time points, potentially diluting time-specific effects and increasing clinical heterogeneity. Another important consideration is the evolution of implant design and surgical technique over time. Many of the older trials included in earlier meta-analyses used older femoral components with less anatomically accurate trochlear geometry [59–63]. Contemporary patellar-friendly designs aim to optimise tracking and reduce contact stress even in the absence of resurfacing [19, 56, 64, 65]. Meta-analyses spanning several decades, therefore, combine fundamentally different biomechanical environments, making it difficult to isolate the true effect of resurfacing independent of implant generation. Most reviews were unable to stratify outcomes according to design features, leaving this source of heterogeneity unresolved. Outcome definitions further contributed to variability. Reoperation was not uniformly defined across meta-analyses. Some considered any return to theatre as a reoperation, whereas others restricted the definition to patellofemoral-related procedures or separated patellofemoral from non-patellofemoral causes. AKP itself represents a heterogeneous endpoint. Across primary trials, it was defined and measured inconsistently, assessed using non-uniform instruments, and treated as either a binary outcome or a graded measure. As previously discussed, given the multifactorial nature of AKP, pooling such heterogeneous assessments is likely to generate inconsistent results. It may partially explain the variable analgesic signals observed across meta-analyses. Although the AMSTAR-2 assessment was performed independently by two reviewers, formal inter-rater reliability statistics were not calculated. Therefore, the consistency of the quality appraisal process cannot be quantified objectively. Subgroup analyses by follow-up duration, implant design, and study characteristics could not be performed because these data were inconsistently reported across the included meta-analyses and were not available in a sufficiently homogeneous format to allow a reliable umbrella-level reanalysis. Although several meta-analyses reported additional outcomes such as the Oxford Knee Score (OKS), Knee Osteoarthritis Outcome Score (KOOS) domains, visual analogue scales (VAS), complication profiles, and economic endpoints, these outcomes were reported inconsistently. They were supported by a limited number of primary studies. Their inclusion as core endpoints in an umbrella review would have introduced selective outcome reporting at the umbrella level. For this reason, they were not included among the primary outcomes of interest. This heterogeneity observed across meta-analyses reflects genuine diversity in patient populations, implant designs, surgical indications, follow-up structures, and outcome definitions. While this heterogeneity limits the ability to draw highly granular conclusions regarding patient selection or implant-specific effects, it does not negate the consistency of the observed association between patellar resurfacing and reduced reoperation rates. Rather, it underscores the need for future studies designed around clearly defined patellofemoral phenotypes and contemporary implant technologies. Reoperation, rather than revision, was selected as the outcome of interest because the included meta-analyses predominantly reported this broader endpoint, encompassing any further surgical procedure related to the patellofemoral joint, including secondary patellar resurfacing, lateral release, debridement, and arthroscopic procedures, in addition to formal component revision. Revision rates were reported inconsistently and infrequently across the primary meta-analyses, which would have substantially limited the number of eligible reviews and the statistical power of the umbrella-level synthesis. This distinction is acknowledged as a limitation, as reoperation and revision differ in clinical severity and implication, and the pooled estimates should be interpreted as reflecting the broader composite endpoint rather than formal implant revision specifically. Finally, the present umbrella review was not prospectively registered, which should be acknowledged as a limitation. Although the review followed predefined eligibility criteria and outcomes, the absence of a registered protocol may have introduced a potential risk of outcome reporting bias and post hoc analytical changes.

## Conclusion

Patellar resurfacing in primary TKA consistently reduces the risk of reoperation across meta-analyses, but provides no clinically meaningful improvement in PROMs and only limited, inconsistent effects on AKP. This pattern suggests that resurfacing primarily reduces the likelihood of secondary surgery rather than enhancing pain relief or function. Any benefit appears restricted to a small subset of patients with relevant patellofemoral pathology and is diluted in heterogeneous populations, particularly with modern patellar-friendly implant designs. Overall, the evidence supports patellar resurfacing as a data-driven approach for risk mitigation, underscoring the need for future indication-based studies using contemporary implants and standardised outcomes. Future trials should prospectively stratify patients according to patellofemoral cartilage status, preoperative anterior knee pain, and implant design. Standardised criteria for selective resurfacing and longer follow-up periods are also needed to determine whether specific patient subgroups derive meaningful benefit from patellar resurfacing.

## Supplementary Information

Supplementary Material 1.

Supplementary Material 2.

## Data Availability

All data generated or analysed during this study are included in this published article.
