# Supplementary material for: Patellar resurfacing in primary total knee arthroplasty is associated with fewer reoperations but comparable clinical and functional outcomes: an umbrella review of meta-analyses
Source: Arthroplasty. 2026 Oct 6;8:75. doi: 10.1186/s42836-026-00438-y (PMC13640065; doi:10.1186/s42836-026-00438-y)
Supplement: Supplementary file 1 [file 42836_2026_438_MOESM1_ESM.docx]

**Supplementary Figure S1.** Radar plot illustrating the AMSTAR-2 item-level profile of a high-confidence review (Grela et al., 2022) compared with a critically low-confidence review (Tang et al., 2018). Items are numbered according to the AMSTAR-2 checklist.
