# Supplementary material for: Patellar resurfacing in primary total knee arthroplasty is associated with fewer reoperations but comparable clinical and functional outcomes: an umbrella review of meta-analyses
Source: Arthroplasty. 2026 Oct 6;8:75. doi: 10.1186/s42836-026-00438-y (PMC13640065; doi:10.1186/s42836-026-00438-y)
Supplement: Supplementary file 2 [file 42836_2026_438_MOESM2_ESM.docx]

| **Item** | **AMSTAR-2 item description** |
| --- | --- |
| 1 | Research questions and inclusion criteria include the components of PICO (Population, Intervention, Comparator, Outcome). |
| 2 | Review methods were established prior to the conduct of the review, including protocol registration. |
| 3 | The authors explained their selection of study designs for inclusion in the review. |
| 4 | A comprehensive literature search strategy was used. |
| 5 | Study selection was performed in duplicate. |
| 6 | Data extraction was performed in duplicate. |
| 7 | A list of excluded studies was provided and reasons for exclusion were justified. |
| 8 | The included studies were described in adequate detail. |
| 9 | The authors used a satisfactory technique for assessing the risk of bias in individual studies. |
| 10 | The sources of funding for the included studies were reported. |
| 11 | Appropriate methods were used for the statistical combination of results, when meta-analysis was performed. |
| 12 | The potential impact of risk of bias in individual studies on the results was assessed. |
| 13 | Risk of bias was taken into account when interpreting and discussing the results of the review. |
| 14 | The authors provided an explanation for, and discussion of, observed heterogeneity. |
| 15 | Publication bias was investigated and its potential impact on the results was discussed. |
| 16 | Potential conflicts of interest and sources of funding for the review were reported. |

**Supplementary Table S1:** Description of AMSTAR-2 items.
